# Specific Pandemic-Related Worries Predict Higher Attention-Related Errors and Negative Affect Independent of Trait Anxiety in UK-Based Students

**DOI:** 10.1007/s10608-022-10336-7

**Published:** 2022-10-20

**Authors:** Chris R. H. Brown, Ya-Chun Feng, Vlad Costin, Colette R. Hirsch, Yun-Han Wang, Yun-Lin Wang, Jowinn Chew, Jordan Kenny, Paul Allen

**Affiliations:** 1 University of Roehampton, London, UK; 2 National Sun Yat-Sen University, Kaohsiung, Taiwan; 3 University of Sussex, Brighton, UK; 4 Kings College London, London, UK; 5 Kings College London, University of Roehampton, London, UK; 6 School of Psychology, Whitelands College, University of Roehampton, Holybourne Avenue, London, SW15 4JD UK

**Keywords:** Quality of life, Anxiety, Attentional control, Worry, Pandemic-related worry, COVID-19

## Abstract

**Background:**

The COVID-19 pandemic has resulted in many individuals experiencing increased symptoms of anxiety. We predict that this increase may be underpinned by pandemic-related worry (PRW), characterised by repetitive negative thinking about pandemic-specific outcomes; and that this relationship is mediated through reduced attentional capacity required to regulate negative affect.

**Methods:**

We developed a novel scale to measure the contents of PRW in an initial sample of 255 participants, and explored its relationship with cognitive functioning and negative affect in a sample of 382 UK-based university students, whilst controlling for recalled pre-pandemic trait anxiety.

**Results:**

A five-factor model of PRW was identified, with factors reflecting worry about decline in quality of life (QoL) and probability of infection correlating with attention and memory-related errors. Importantly, attention-related errors partially mediated the positive relationship between PRW and negative affect, even when controlling for pre-pandemic trait anxiety.

**Conclusion:**

PRW’s relationship with negative affect was partially mediated through attentional function, consistent with models of anxiety and attentional control. In UK-based students PRW may be predominantly focused on the decline in QoL; therefore, interventions targeting worry about the decline in QoL caused by COVID-19 are especially important in this population in the wake of the pandemic.

**Supplementary Information:**

The online version contains supplementary material available at 10.1007/s10608-022-10336-7.

## Introduction

As well as the direct risk to physical health, the COVID-19 pandemic has had an adverse effect on mental health across the world (Pfefferbaum & North, 2020). Research identifying risk factors for this decline in mental health have found that those with pre-existing psychiatric conditions are at greater risk of increased anxiety and depression (Asmundson et al., 2020; Fancourt et al., 2021; Porter et al., 2021). However, evidence has also suggested that mental health issues during the pandemic are not limited to those who were already experiencing anxiety and depression. For instance, studies comparing to pre-pandemic baseline measures have found an increase in depression, anxiety, and worry even in individuals without pre-existing mental health conditions (Kwong et al., 2021; Pan et al., 2021; Ravens-Sieberer et al., 2021b).

Whilst the rise in anxiety may be temporary in some cases, for many the experience of anxiety can become long lasting and uncontrollable. Research exploring the development of anxiety suggests that the transition from the dispositional vigilance for potential threat to chronic anxiety is dependent upon the ability to regulate worry; that is, repetitive negative automatic thoughts about anticipated threats (Hudson & Rapee, 2004; Lahat et al., 2011; Perez-Edgar & Fox, 2005). Indeed, longitudinal investigations have found that increased worry at earlier timepoints can predict a subsequent increase in anxiety that is independent of baseline levels of anxiety (Calvete et al., 2013; Spinhoven et al., 2018). Thus, the widespread increase in pandemic-related worry (PRW) beyond what is normally experienced by individuals without anxiety may precede the emergence of more severe anxiety disorders in the population.

Models of cognitive-emotional interactions propose that worry reflects the cognitive component of anxiety, and that as well as resulting in elevated negative affect (Calmes & Roberts, 2007), worry occupies the limited cognitive capacity required to complete tasks that require attentional/cognitive resources (Eysenck et al., 2007; Hirsch & Mathews, 2012). This interference can result in less efficient task performance, lapses in concentration, and increased mind-wandering. For instance, experimental evidence shows that inducing worry about a negative emotional event results in reduced working memory capacity, relative to when thinking about positive events, as indexed by increased error rates during behavioural working memory tasks (Hayes et al., 2008; Leigh & Hirsch, 2011; Sari et al., 2017; see Moran, 2016, for review).

Due to this interference with attentional capacity, the ability to regulate worry becomes difficult as the disengagement from worrying thoughts requires top-down control (Hirsch & Mathews, 2012). As such, elevated worry can become self-perpetuating with reduced attentional capacity predicting elevated worry, which in turn predicts reduced attentional capacity (Cox et al., 2018; Trezise & Reeve, 2016). Based on limited cognitive capacity models of anxiety, it would be expected that elevated levels of worry during the COVID-19 pandemic would reduce attentional capacity, which in turn could result in continued worrisome thoughts and higher negative affect.

Several investigations have reported a negative correlation between COVID-19 specific measures of anxiety and cognitive function. For instance, a novel measure of fear of COVID-19 infection (i.e. fear of coronavirus scale; Ahorsu et al., 2020) was found to negatively correlate with behavioural measures of executive functioning such as task-switching and processing speed (da Silva et al., 2021). Similarly, Fellman et al. (2020) found that a single item measuring the general increase in anxiety due to the pandemic was correlated with poorer working memory performance using the n-back task. Podlesek et al. (2021) also found that single item measures of pandemic-related stress at work or home independently correlated with poorer self-reported cognitive functioning.

These findings provide evidence for the hypothesis that PRW may uniquely interfere with cognitive functioning. These investigations, however, only took a narrow focus by using single-item scales or questionnaires focused on the direct fear elicited by COVID-19. Given the widespread impact of the COVID-19 pandemic on different aspects of life, it is likely that PRW encompasses more than the direct fear of COVID-19, and that some PRWs will be more frequent and disruptive to cognitive functioning than others. Additionally, though these previous scales identify core concerns, they do not measure the full range of probable topics of worry. For example, the worry domains questionnaire developed by Tallis et al. (1992) encompasses five common topics of worry, these being worries about relationships, academic and occupational work, financial concerns, worries about the future, and lack of confidence. These factors are underrepresented in existing pandemic scales, despite likely being heightened in the pandemic beyond baseline levels. For instance, social isolation would increase worry about social relationships, and economic disruption would heighten financial concerns.

The current investigation therefore aimed to identify the specific factors of PRW, and which specific PRWs were most strongly correlated with attention and memory-related errors, as well as their relationship with recent negative affect (i.e. depression, anxiety, and stress symptoms). The current investigation will therefore address three key aims, (1) to identify the contents and factor structure of PRW; (2) test in a cross-sectional design which specific PRW factors are most strongly correlated with deficits in self-reported attention and memory functioning; and (3) explore whether the relationship between PRW and higher recent negative affect is mediated through reduced attentional capacity (i.e. higher attention-related errors), as hypothesised by influential cognitive-emotional theories of anxiety and worry (Eysenck et al., 2007; Hirsch & Mathews, 2012). Additionally, we propose that PRW reflects a novel phenomenon experienced by many people without a predisposition to habitual worry (Pan et al., 2021). We therefore expect that any relationship between PRW and cognitive errors will remain even when controlling for the recalled baseline pre-pandemic levels of trait anxiety and worry.

## Study 1

### Methods

#### Participants

Two hundred and fifty-five (255) participants were pooled from 3 separate investigations run between May and July 2020. The three samples were recruited from three separate populations: undergraduate students (n = 49), postgraduate trainee teachers (n = 186), and participants from the general public (n = 20). The sample consisted of 55 male and 200 female participants, the mean age was 28.02 (*SD* = 10.28). Participants were aged 18 or over, and were recruited from a range of sources including the King’s College London (KCL) university student participant pool, as well as online adverts, and posters. The incentive for the majority of participants was the opportunity to win gift vouchers ranging between £50 and £200, though some participants were renumerated with £15 payment, or completed the study on a voluntary basis. Participants all provided informed consent prior to completion of the study.

#### Materials

##### Pandemic-Related Worries Questionnaire (PRWQ)

Participants completed the initial 30-item version of the PRWQ questionnaire, which measured a range of different concerns relating to the pandemic. To cover the full breadth of potential worries that could be experienced the items were generated for areas which consistently emerge as common topics of worry, as previously identified in the Worry Domains Questionnaire (Tallis et al., 1992). This measure identifies worries from across 5 key domains, including worries about social relationships, academic and occupational performance, financial concerns, disrupted or aimless future goals, and lack of confidence. Items were also generated for two other key areas which are often identified as topics of worry in trait anxiety research, these being worries about physical harm/illness to self and others, and generalised abstract worry (Dugas et al., 1998; Ogniewicz et al., 2014; Roemer et al., 1997). Pandemic-related items were generated for each of these domains by one of the authors (Y.F.) and were discussed with and reviewed by other members of the research team (C.H., J.C., Y-L.W., Y-H.W.). Thus, rather than focusing specifically on the direct worry about the virus, all aspects of PRW were represented in novel the PRWQ.

The response to each item was along a Likert Scale ranging from *‘*Strongly disagree’ (1) to ‘Strongly agree’ (5)*,* for how they felt over the past month. The full PRW-30 items are listed in Table 1, with the finalised PRWQ-20 scale listed in the Appendix which also contains the complete instructions, and all items and subscales finalised after factors had been extracted and confirmed.

**Table 1:** List of all items in their factors derived from the Exploratory Factor Analysis (N = 255)

Item number	Item	Decline in QoL	Infection severity	Risk to loved ones	Infection probability	Financial concerns
**24**	**I often worry that the lockdown will influence my own physical and/or mental health**	**0.775**				
**30**	**I often worry that the isolation situation will influence my relationship with people I live with in a bad way**	**0.673**				
**26**	**I often worry that things will not get back to normal**	**0.648**				
**9**	**I often think about the impact of social distancing and how this will affect me**	**0.612**		− 0.231		
**19**	**I often worry that my relationships will be impacted due to COVID -19 pandemic**	**0.567**				
25	I often worry that the lockdown will influence the physical and/or mental health of someone I am close to (e.g., family, close friends)	0.544		− 0.284		
**28**	**I often worry that I am not performing well in work/study during the lockdown**	**0.519**				
**18**	**I often worry that my work/academic performance will be impacted due to COVID -19 pandemic**	**0.509**				
27	I often worry that I am not taking the right actions to prevent the spread of COVID -19	0.414			− 0.220	
15	I often worry about the consequence of COVID-19 for my close friends	0.358		− 0.260		
**20**	**I often worry that if I get COVID -19 I will not recover from it**		**− 0.895**			
**22**	**I often worry that I will get hospitalised or will die due to COVID -19**		**− 0.812**			
**21**	**I often worry that if my close friend(s) or family member(s) contracted COVID -19, they will not recover from it**			**− 0.818**		
**5**	**I often worry about my family members getting COVID-19**			**− 0.802**		
**11**	**I often worry that someone I love (e.g., family, close friends) will get infected by COVID-19 virus**			**− 0.769**		
**23**	**I often worry about whether my close friend(s) or family member(s) will be hospitalised or die due to COVID -19**			**− 0.740**		
14	I often worry about whether my close friend(s) or family member(s) will be hospitalised or die due to COVID -19	0.253		− 0.517		
**3**	**When I learn or read about COVID-19 I become worried that I may have it**				**− 0.830**	
**1**	**I often worry about the possibility that I have COVID-19**				**− 0.828**	
**2**	**I get concerned when I experience symptoms in case I have COVID-19**			− 0.206	**− 0.616**	
10	I often worry that I will get infected by COVID-19 virus		− 0.244		− 0.531	
**4**	**If COVID-19 is brought to my attention (through the radio, television, newspapers, or someone I know), I worry about getting it myself**				**− 0.495**	
17	I often worry that I will not get everyday necessities like food and other grocery items due to COVID-19 pandemic	0.240			− 0.269	
**8**	**I often worry about the impact of COVID-19 on my financial situation**					**− 0.845**
**7**	**Hearing about job losses in the media or through people I know makes me worry about my job security**					**− 0.833**
**12**	**I often worry about the impact of COVID-19 on my family’s financial situation**					**− 0.524**
29	I often worry that my future job/study prospects will be influenced by COVID-19 pandemic	0.294				− 0.485
16	I often worry that I will not achieve something important to me due to COVID-19 pandemic	0.337				− 0.377
6	The COVID-19 pandemic makes me worry about the future	0.315		− 0.259		− 0.320
13	I often worry about the consequence of COVID-19 for myself	0.257	− 0.211		− 0.236	− 0.288
	Eigenvalues	12.88	2.55	1.76	1.45	1.12
	Percentage variance	42.95%	8.50%	5.88%	4.82%	3.75%
	Cronbach’s α / Spearman’s *r*	.853	*r* = .78	.923	.879	.834
	Items	7	2	4	4	3
	Mean score (SD)	3.50 (1.21)	2.37 (1.18)	3.78 (1.13)	2.70 (1.20)	3.34 (1.31)

Items loading uniquely on a factor are highlighted in bold. Only loadings with an absolute value greater than .2 are displayed for visibility

#### Procedure

Participants reported demographic information, including sex (male, female) and age. Participants completed the PRWQ, which was included as a measure in three separate testing sessions of projects exploring cognitive-emotional factors in mental health, conducted as part of doctoral thesis projects.

### Results and Discussion

The data were analysed using exploratory factor analysis in JASP software (JASP team, 2021). This was conducted using a maximum likelihood estimation, and direct oblimin rotation due to expected correlating factors. Based on guidelines outlined in Costello and Osborne (2005), items were only retained if their loading on a factor was greater than .3. Cross-loading items were retained on the factor they loaded most strongly on, but only if the difference between absolute loadings was greater than .3 (see Table 1). The number of factors extracted was based on a combination of scree plot and parallel analysis. The parallel analysis was used to compute the 95^th^ quantile eigenvalue estimates, which were then superimposed on the scree plot to facilitate the identification of the breakpoint. Inspection of this scree plot revealed that there were five factors which were above the simulated 95^th^ quantile cut-off (see Supplementary materials 1). Additionally, these five potential factors were the only factors with eigenvalues greater than 1.

Assessing the contents of the five factors revealed that they reflected the perceived decline in quality of life due to the pandemic (“decline in QoL”), the potential risk of severe COVID-19 infection (“infection severity”), the risk of COVID-19 to family and friends (“risk to loved ones”), the probability that they may be infected with COVID-19 (“infection probability”), and financial stress caused by the pandemic (“financial concerns”; see Table 1 for all items and factor loadings).

In comparison, a potential four-factor model revealed a similar pattern, with the exception that the two factors related to risk from COVID-19 (i.e. infection probability, and infection severity) loaded on a single COVID-19 risk factor (see Supplementary materials 2 for four factor solution). Based on evidence that beliefs about COVID-19 infection probability and COVID-19 infection severity worries predict different emotional and behavioural outcomes (e.g. smoking behaviour, post-traumatic stress response, and attentional biases; Brown, 2021; Canitto et al., 2020; Di Crosta et al., 2020), merging these factors could obscure similar differences in the current data. We therefore selected the five-factor model.

The decline in QoL was the factor which accounted for the most variance in the data (43%). This is consistent with the findings that young adults, who were the majority of our sample, reported a decline in QoL during the COVID-19 pandemic across several international samples, specifically in areas of physical, psychological, social and environmental domains of QoL (Abdullah et al., 2021; Azzi et al., 2021; Ravens-Sieberer et al., 2021a; Szczepańska & Pietrzyka, 2021).

Interestingly, the financial concerns factor loaded on a separate factor from decline in QoL despite financial security often being a measure of QoL (Michalos & Kahlke, 2010). This finding suggests that financial worries weren’t integrated into experiences of QoL for the current sample, potentially due to the sample being university students, whose income may be based more on government loans and family support than from regular employment (Bolton, 2021).

In previous investigations, the direct fear of infection has been the primary factor identified in the factor analysis, which loads on a single COVID-19 risk factor (Taylor et al., 2020). Conversely, in the current investigation the risks from COVID-19 loaded on three separate factors reflecting COVID-19 infection probability, the COVID-19 infection severity, and the risk from COVID-19 to loved ones. The potential reason for this could be due to greater variation in the contents of the initial items relative to earlier scales (e.g. Taylor et al., 2020), allowing more variation in response. Alternatively, the findings could reflect the younger age of the current participants relative to the previous sample. The average age of the sample recruited by Taylor et al. was 49.8, whilst the average age of the current sample was 28.02. The current sample would therefore be less at risk from severe COVID-19 symptoms but still prone to infection, potentially explaining the different factor loadings.

We note that the current scale was developed in a younger student sample, and may not contain the full range of specific worries experienced in an older non-student population, such as concerns about childcare (Racine et al., 2021), or increased occupational workload during the pandemic (Yang et al., 2021). These would, however, likely be correlated with the decline in QoL factor, specifically items reflecting difficulties in relationships at home or performance in work/academic settings.

## Study 2

The aims of Study 2 were to validate the factor structure of the PRWQ in a separate sample, and to assess the relationship between PRW, cognitive functioning, and negative affect during the pandemic. In Study 2 we recruited a new independent sample who completed the PRWQ alongside other measures (see below).

### Methods

#### Participants

Initially, 447 participants responded to the survey link. Inclusion criteria required that participants be aged 18 or over, and were a university student. Participants were excluded if they did not complete the experimental session, provided contradictory responses to COVID-19 diagnosis questions (i.e. a positive test, but reporting not ever having COVID-19), or showed evidence of repetitive responding. Repetitive responders were identified by recording the number of times a participants’ response was identical to a previous item worded in the opposing direction. Those who scored over 2 SDs on this measure were excluded. This data screening protocol is based on the longstring data-screening method (DeSimone et al., 2015); however, this adaptation (designed for the current study) focused more on repeated implausible responses rather than all repeated responses, and was designed to exclude clear cases of unmotivated responding without relying on subjective judgment. The final sample consisted of 382 participants with the mean age of 21.24 (*SD* = 4.95, Range 18–72).^1^ In total there were 313 female participants, 61 male participants, 5 who reported identifying as neither male/female or non-binary, and 3 who preferred not to report. Participants all provided informed consent prior to completion of the study.

Within the sample, 88 participants reported being certain or almost certain they had been infected with COVID-19, of which 58 reported a positive test.^2^ Twelve reported asymptomatic cases, 70 mild illness, 4 a moderate illness requiring hospitalisation, and 1 reporting a severe illness requiring hospitalisation.

Participants were recruited across three different universities (Roehampton, Sussex, KCL) by volunteer mailing list or in exchange for course credit. All Study 2 data was collected between March and May 2021. The recruitment strategy was to recruit as many participants as possible in a single academic term. The final sample was consistent with guidelines for factor analysis which recommend a minimum sample of 300 (Comrey & Lee, 1992), as well as those recommending a minimum number of participants per item (e.g. 10:1 ratio; Osborne & Costello, 2004). Additionally, the average item loading on each factor from Study 1 was .71 (*SD* = .13), and the average number of items per factor was 4 across the five factors; therefore, according to recommendations for Confirmatory Factor Analysis (CFA) by Wolf et al. (2013), the final sample size would be sufficient to validate the factor structure found in our initial sample, based on expected loadings.

#### Materials

##### Depression, Anxiety, & Stress Scale (DASS-21)

To measure recent negative affect over the past month, participants completed the Depression, Anxiety, and Stress Scale (DASS-21; Lovibond & Lovibond, 1995). This is a 21-item scale which measures the frequency that participants experience depression, anxiety, and stress, with each subscale composed of 7 items. The total scale score can be combined to measure overall negative affect. Responses are made along a four-point Likert scale ranging from ‘Never’ (score 0) to ‘Almost always’ (score 3), referring to the past month. Cronbach’s alphas across the for depression, anxiety and stress were .91, .79 and .85, respectively, with the total α = .93. The mean score across subscales for depression, anxiety, and stress were 9.28 (*SD* = 4.34), 6.5 (*SD* = 4.01), and 9.35 (*SD* = 5.16), respectively, with the average total score being 25.13 (11.86).

##### Pandemic-Related Worry Questionnaire (PRWQ)

Participants completed the full 30-item PRWQ used in Study 1. The only change from this initial questionnaire was that the phrase ‘lockdown’ was replaced with ‘government Covid-19 rules (e.g. limited socialising)’, due to the recent lifting of the UK lockdown at the time of testing. Cronbach’s alphas across the subscales were thus: decline in QoL α = .86, infection severity 2-items Spearman’s *r* = .76, risk to loved ones α = .90, infection probability α = .89, financial concerns α = .76, with the total α = .94. The mean scores across subscales were: Decline in QoL = 3.94 (SD = .82), infection severity = 2.33 (*SD* = 1.16), risk to loved ones = 3.72 (*SD* = 1.03), infection probability = 2.65 (*SD* = 1.04), financial concerns 3.51 (*SD* = .98), with the total PRWQ average score = 3.23 (*SD* = .82).

##### Memory Failures Scale (MFS) and Attention-Related Cognitive Errors Scale (ARCES)

To measure recent cognitive functioning over the past month, participants completed the MFS and the ARCES both developed by Carriere et al. (2008). These scales are designed to measure independent aspects of cognitive functioning. The MFS is designed to measure failures to recall semantic information and prospective goals from long-term memory, independent of attention; whilst the ARCES measures the ability to concentrate on a current task, switch attention between tasks, and avoid distraction. Both scales consist of 12 items, each item is rated along a five-point scale ranging from “never” (1) to “very often” (5), referring to the last month. The internal consistency was high for both scales, MFS α = .87; ARCES α = .90. The mean total score for the MFS was 31.61 (*SD* = 8.35), and ARCES was 37.09 (*SD* = 8.99).

##### Trait General Anxiety Disorder (GAD-7)

To measure self-reported trait anxiety prior to the pandemic, participants were asked to complete the 7-item GAD-7 scale (Spitzer et al., 2006), scored along a 4-point Likert scale ranging from “Not at all” to “Almost every day”. Participants were instructed to “*Please answer related to how you have felt MOST OF THE TIME BEFORE THE COVID-19 PANDEMIC, rather than during the Covid-19 pandemic.”* The Cronbach’s alpha for this measure was, α = .91, and the mean score was 8.06 (SD = 5.18).

##### Trait Penn State Worry Questionnaire (PSWQ)

To measure self-reported trait worry prior to the pandemic, participants were asked to rate 16 items of the PSWQ along a 5-point Likert scale (Meyer et al., 1990), ranging from “not at all typical of me” (1) to “very typical of me” (5). To measure pre-pandemic worry, participants were given the same instructions as the GAD-7. The Cronbach’s alpha for this measure was, α = .94, and the mean score was 56.31 (SD = 13.86).

#### Procedure

Participants first provided informed consent, before completing demographic information, which consisted of their age, their gender (male, female, neither male nor female, or prefer not to answer), their ethnicity using categories utilised by the UK office of National Statistics (2022), and their student status and degree. Open response boxes were provided for both gender and ethnicity, in addition to pre-defined categories.

Participants then completed questions referring to COVID-19 infection. These included whether they had ever tested positive for COVID-19 or whether they believed they had ever had COVID-19 (responses ranged from “Certain” to “Impossible”). If participants reported they were ‘almost certain’ they previous had COVID-19, they reported the severity of illness ranging from ‘asymptomatic’ to ‘critical, requiring hospitalisation’, and selected their symptoms from a list of 16 common symptoms (see Sudre et al., 2020). Participants then completed the DASS-21, PRWQ, MFS, ARCES, PSWQ, and GAD-7. Finally, participants then completed items for exploratory analysis relating to substance use, racial discrimination, and disease stigma experienced during the pandemic. The full survey including individual items, and data are available via the OSF (OSF link: https://osf.io/k8qna/?view_only=6a7dc519471349afb2eb60df5a7b9bcc).

#### Results and Discussion

To validate the five-factor model of the PRWQ (see Study 1 above), we conducted CFA which was conducted using a maximum likelihood estimator using JASP software. This analysis found an adequate fit to the data with the previous five-factor model (Hu & Bentler, 1999), *χ*^*2*^(df = 160) = 568.58, *p* < .001 CFI = .908. RMSEA = .082 (90CI[.075, .089]), SRMR = .051 (see Table 2).

**Table 2:** Confirmatory factor analysis results, with parameter estimates, p-values, and 95% Confidence Intervals (95CI) for the five-factor model

Factor	Item number	Parameter estimate	SE	Z	P-value	95CI Lower	95CI Upper
Decline in QoL	24	.772	.045	17.175	< .001	.684	.86
	30	.593	.049	12.104	< .001	.497	.689
	26	.685	.047	14.558	< .001	.593	.777
	9	.728	.046	15.817	< .001	.638	.819
	19	.619	.048	12.778	< .001	.524	.714
	28	.749	.046	16.442	< .001	.66	.838
	18	.705	.047	15.127	< .001	.614	.796
COVID infection severity	20	.893	.044	20.329	< .001	.806	.979
	22	.87	.044	19.643	< .001	.783	.957
Risk to loved ones	21	.865	.042	20.718	< .001	.783	.947
	5	.737	.045	16.327	< .001	.648	.825
	11	.795	.044	18.215	< .001	.709	.88
	23	.895	.041	21.892	< .001	.815	.975
COVID infection probability	3	.837	.043	19.589	< .001	.753	.921
	1	.866	.042	20.65	< .001	.784	.948
	2	.798	.044	18.24	< .001	.713	.884
	4	.785	.044	17.774	< .001	.698	.871
Financial concerns	8	.824	.047	17.514	< .001	.732	.917
	7	.704	.049	14.386	< .001	.608	.8
	12	.656	.05	13.175	< .001	.558	.753

Conversely, CFA on the alternative four-factor solution identified in Study 1, which combined worries about infection severity and infection probability within a single factor, revealed a poorer fit to the data, χ2(df = 183) = 836.42, p < .001; CFI = .867; RMSEA = .097 (90CI[.09, .103]), SRMR = .06. Indeed, a nested model comparison suggested that the five factor model had significantly better fit compared to the four factor model, Δχ2(23) = 267.84, p < .001.

Whilst the five-factor model was adequate, examination of the modification indices (MI) and expected parameter change (EPC) values revealed it was possible that a six-factor model could have been missed. Allowing the errors of the item pair with the highest modification index (MI = 90.56, EPC = .24; items 5 and 11) to covary in the model did somewhat improve the model fit, χ2(df = 159) = 478.55, *p* < .001; CFI = .928; RMSEA = .073 (90CI[.065, .08]), SRMR = .053, and this improvement was significant relative to the unadjusted five-factor model, Δ*χ*^*2*^(1) = 90.03, *p* < .001. This pair of items reflected the worries about the probability that a loved one may be infected with COVID-19; whilst the remaining 2 items within the ‘risk to loved ones’ factor reflected infection severity. It may be, therefore, that the severity risk worries and infection risk worries for loved ones reflect distinct factors which are missed due to their under-representation in the initial pool of items in the PRWQ. Any future versions of the PRWQ may therefore require additional items when exploring worries about the risk to loved ones, specifically. As noted below, however, worries about risk to loved ones were more weakly correlated with cognitive outcomes relative to other factors within the scale, thus the five-factor model is valid in the current context.

One possible confounding factor to be considered could be the development of vaccines, which could alter the level of different pandemic-related worries. In the UK, the vaccination programme began in December 2020, after Study 1 but prior to Study 2. This is however unlikely to be a substantial influence on perceptions of personal risk from COVID-19, as many of the participants were ineligible for vaccination during the March–May 2021 recruitment period, due to those below the age of 30 (96.6%; N = 369) only becoming eligible for vaccination in June 2021. Although it is possible that worries about risks to older loved ones could have been reduced, our data revealed that there was no difference between Study 1, when no vaccine had been approved, and Study 2, when it was approved but not widely available, on any of the subscales related to the risk of infection from COVID-19 to self or loved ones, Cohen’s *d* < .05, *t* < .64, *p* > .521. We would, however, expect that widespread vaccination would likely have reduced worries about severity and probability of infection due to its efficacy (Sadoff et al., 2021), but also resulted in novel worries about side-effects, encountering unvaccinated individuals, or worries about mandatory vaccination (Bendau et al., 2021).

Though worries about the direct threat of COVID-19 did not differ between Study 1 and 2, worries about QoL were significantly lower in Study 2, *d* = − .52, *t*(515.28) = 6.38, *p* < .001. With worries about financial concerns also showing a similar pattern, albeit weaker and failing to reach significance, *d* = − .16, *t*(486.97) = 2.01, *p* = .052. This difference is consistent with Study 1 occurring during full lockdown, and Study 2 occurring after lockdown was lifted (with restrictions in place) when QoL concerns would be less salient. It could however also reflect a general increase in optimism about the course of the pandemic caused by vaccine development. Importantly, the factor structure of PRW was replicated across both Study 1 and Study 2, suggesting a limited overall change in the relational structure between factors between the two timepoints.

#### The Relationship Between PRW and Cognitive Functioning

As predicted, zero-order correlations (see Table 3) revealed a significant positive correlation between both the MFS (assessing memory) and ARCES (assessing attention) and all subscales of the PRWQ, which were in line with the probable effect size based on meta-analytic estimates of the relationship between anxiety and cognitive function (*r* = .28; Shi et al., 2019). All other variables including all DASS subscales, and pre-pandemic trait anxiety and worry, measured with GAD-7 and PSWQ, also positively correlated with all PRWQ subscales.

**Table 3:** Zero-order correlations between the Pandemic-Related Worries Questionnaire (PRWQ) and identified subscales, Attention-Related Cognitive Errors Scale (ARCES), Memory Failures Scale (MFS), Depression, Anxiety, and Stress Scale (DASS) scores

		1	2	3	4	5	6	7	8	9	10	11	12	13
1	PRWQ decline in QoL	–												
2	PRWQ infection probability	.31***	–											
3	PRWQ infection severity	.26***	.59***	–										
4	PRWQ risk to loved ones	.44***	.54***	.50***	–									
5	PRWQ financial concerns	.58***	.34***	.38***	.46***	–								
6	PRWQ total	.66***	.77***	.77***	.80***	.73***	–							
7	Memory-related errors (MFS)	.25***	.31***	.23***	.21***	.20***	.32***	–						
8	Attention-related errors (ARCES)	.37***	.29***	.25***	.25***	.25***	.37***	.75***	–					
9	Depression (DASS)	.47***	.15*	.28*	.22***	.28***	.32***	.36***	.43***	–				
10	Anxiety (DASS)	.37***	.29***	.31***	.32***	.33***	.43***	.37***	.44***	.59***	–			
11	Stress (DASS)	.46***	.28***	.21***	.29***	.31***	.40***	.38***	.47***	.72***	.65***	–		
12	Negative affect (DASS total)	.50***	.27***	.30***	.31***	.34***	.43***	.42***	.51***	.90***	.83***	.90***	–	
13	Trait anxiety (pre-pandemic recall)	.32***	.29***	.24***	.23***	.23***	.37***	.43***	.47***	.47***	.55***	.59***	.59***	–
14	Trait worry (pre-pandemic recall)	.36***	.32***	.24***	.28***	.21***	.37***	.28***	.34***	.43***	.47***	.55***	.55***	.69***

Negative affect reflects the combined DASS scale score

***p < .001; *p < .05. All p-values were adjusted using the Holm correction for multiple comparisons (Holm, 1979)

To explore which subscales of the PRWQ were most strongly associated with attention and memory functioning, we entered all five PRWQ subscales into two separate regression models with ARCES scores and MFS scores as separate outcome variables (see Table 4). Interestingly, two of the PRWs were independently associated with MFS and ARCES scores, these were the worries about the decline in QoL and the worries about infection risk. All other subscales became non-significant when simultaneously entered into the model.

**Table 4:** Linear regression analyses for Attention-related errors and Memory-related errors with all Pandemic-Related Worries Questionnaire (PRWQ) subscales as predictor variables

		Attention-related errors						Memory-related errors					
		β	*t*	*p*	95% CI LB	95% CI UB	unique *R*^*2*^	β	*t*	*p*	95% CI LB	95% CI UB	unique *R*^*2*^
Step 1—PRWQ subscales	**Decline in QoL**	**.30**	**5.11**	** < .001**	**.19**	**.42**	**.082**	**.17**	**2.81**	**.005**	**.05**	**.29**	**.032**
	**Infection probability**	**.15**	**2.36**	**.019**	**.03**	**.27**	**.035**	**.22**	**3.45**	**.001**	**.10**	**.35**	**.049**
	Infection severity	.10	1.56	.119	− .03	.22	.022	.06	.98	.298	− .06	.19	.018
	Risk to loved ones	− .01	.17	.868	− .13	.11	.02	− .02	− .32	.727	− .15	.11	.012
	Financial concerns	< .01	< .01	.999	− .12	.12	.017	.01	.11	.918	− .12	.13	.011
		*R*^*2*^ = .176, *F*(5,376) = 16.07, *p* < .001						*R*^*2*^ = .121, *F*(5,376) = 10.36, *p* < .001					
Step 2—trait covariates	**Decline in QoL**	**.22**	**3.76**	** < .001**	**.10**	**.33**	–	.10	1.59	.114	− .02	.21	–
	Infection probability	.10	1.70	.090	− .02	.21	–	**.18**	**2.95**	**.003**	**.06**	**.30**	–
	Infection severity	.04	.63	.532	− .08	.15	–	< .01	.06	.951	− .11	.12	–
	Risk to loved ones	< .01	< .01	.940	− .11	.12	–	< .01	.06	.956	− .12	.12	–
	Financial concerns	< .01	.03	.979	− .11	.11	–	< .01	.09	.932	− .11	.12	–
	Pre-pandemic trait anxiety	.39	6.29	< .001	.27	.51	–	.40	6.19	< .001	.27	.52	–
	Pre-pandemic trait worry	− .04	.69	.491	− .17	.08	–	− .08	− 1.28	.201	− .21	.05	–
		*R*^*2*^ = .286, *F*(7,374) = 21.44, *p* < .001						*R*^*2*^ = .225, *F*(7,374) = 12.49, *p* < .001					

Variables which significantly predict attention or memory-related errors are highlighted in bold. Unique *R*^*2*^ refers to unique contribution to the overall *R*^*2*^ by the variable relative to other variables, as calculated using dominance analysis

We note however that the PRWs were highly correlated, meaning that variation in one PRW may be dependent upon changes in another PRW subscale, making it difficult to determine the relative associative strength with the outcome variable. To address this, we supplemented the results with dominance analysis, which assesses the relative explanatory power of one variable over another when every combination of predictor variable is analysed (Budescu, 1993). This was conducted using the R *dominanceanalysis* package (Navarrete & Soares, 2020). We first calculated the average unique contribution to the *R*^*2*^ of attention and memory-related errors by each predictor variable, relative to competing variables in the model (see Table 4, ‘unique *R*^*2*^’ columns). This replicated the pattern of importance found in the linear regression. To assess the generalisability of the dominance structure, we then assessed the proportion of times the dominance structure was reproduced across 5000 bootstrapped samples (Azen & Budescu, 2003).

When assessing the strength of the unique associative contributions to attention-related errors (ARCES score), we found that worries about decline in QoL completely dominated all other PRWs in 78.4% to 99.3% of bootstrap samples; and worries about COVID infection probability completely dominated all other PRWs in 47.4% to 67.5% of bootstrap samples. No other variable completely dominated another variable in the model. For memory-related errors (MFS score), we found worries about COVID-19 infection probability completely dominated decline in QoL in 53.1% of bootstrapped samples and all other variables in 90.1% to 94.6% of bootstrap samples. Decline in QoL then dominated all other PRWQ subscales in 50.9% to 88.7% of bootstrap samples. Though worries about COVID-19 severity dominated risk to loved ones in 39.4% of samples, there was no clear pattern of dominance across the other comparisons (see Supplementary materials 3 for bootstrapped dominance analysis tables). The dominance analysis therefore confirmed findings from the linear regression suggesting that decline in QoL and COVID-19 infection probability were the factors most strongly correlated with cognitive function.

The significant relationship between worry about declining QoL and cognitive functioning is consistent with evidence that young adults are highly concerned about the indirect impact of the pandemic on their mental health and social life, more than about the direct threat from the virus itself (Groake et al., 2021; Ranta et al., 2020). The reason that worries about infection probability was correlated with cognitive functioning more than the infection severity worries may be because the sample were predominantly young adults, and would be less at risk from severe infection, but no less likely to be infected (Davies et al., 2020). On the other hand, it could be that probability of infection may be an indirect measure of the current state of the pandemic at the time of report. As cases increase in the population, so would the probability of infection, meaning that probability of infection could also reflect concerns about the overall progression of the pandemic and its subsequent impacts.

To explore whether the relationship between PRW and attention and memory-related errors was independent of pre-existing trait anxiety and worry, these measures were entered into the regression model (Table 4). When added to the model, recalled trait anxiety was the primary predictor of both attention and memory-related errors, above recalled trait worry or any of the PRWQ subscales, consistent with anxiety linked impairments in executive functioning (Eysenck et al., 2007; Moran, 2016).

To further explore the proportion of unique variance accounted for by PRW, independent of trait anxiety and worry, the order that the trait covariates and PRWQ subscales were entered into the model was reversed, to assess the change in *R*^*2*^. The trait measures accounted for 22.1% of the variance in attention-related errors, and 18.1% of the variance in memory-related errors when entered in step 1 (*p*’*s* < .001). The entry of the PRWQ subscales in the model did however result in a significant increase in *R*^*2*^, revealing that PRW accounted for an additional 6.6% of the variance in attention-related errors, *R*^*2*^_*change*_ = .066, *F*(5, 374) = 6.89, *p* < .001, and 4.4% of the variance in memory-related errors, *R*^*2*^_*change*_ = .044, *F*(5, 374) = 4.23, *p* = .001, independent of trait anxiety and worry. The independent relationships are consistent with the hypothesis that individuals without pre-existing high levels of trait anxiety or worry may have experienced interference from PRW on cognitive functioning.

In order to determine whether PRW correlated with both attention and memory function independently, or whether their relationships reflect the same underlying process, we conducted an exploratory follow-up partial correlation analysis. The relationship between total PRWQ score and memory-related errors, whilst controlling for attention-related errors, was found to be non-significant, *r*(379) = .06, *p* = .26; conversely, the relationship between attention-related errors and total PRWQ score remained significant when controlling for memory-related errors, *r*(379) = .22, *p* < .001. Thus, attention-related errors were more strongly related to PRW than memory-related errors, with the MFS likely correlating with PRW due to shared variance with attention-related functioning; either due to a common relationship with general cognitive ability, or attention-related functioning’s partial role in long-term memory retrieval (Kane & Engle, 2000; Unsworth, 2010). The current findings are consistent with anxiety disrupting attention and executive function, rather than the recall of information from long-term memory (Eysenck et al., 2007).

The ARCES measures the frequency of cognitive errors in real-world situations, such as losing concentration whilst reading, zoning out during conversations, increased interference whilst multitasking, and distractibility. Increased inattention in these situations can result in a wide range of negative outcomes, for instance in occupational settings the elevated inattention would likely result in poorer work performance which could contribute to job insecurity and stress; and more broadly, constitute a hidden economic cost caused by the pandemic (Cutler & Summers, 2020).

For students, who made up our second sample and most of our first sample, PRW may pose a direct threat to their ability to perform academically. For instance, worry has been linked to reduced academic performance over time, with earlier levels of worry predicting subsequent lower academic achievement (Owens et al., 2012). PRW may reduce academic achievement both through the ability to learn, by reducing the ability to focus in lectures and when reading (Risko et al., 2012; Unsworth et al., 2013), as well as performance in assessment situations, where current concerns increase off-task thoughts (Jordano & Touron, 2017; Mrazek et al., 2011). The novel PRW may therefore result in poorer academic performance during the pandemic due to its disruption of attentional mechanisms.

#### The Mediating Role of Attention-Related Errors Between PRW and Negative Affect

**Fig. 1:**
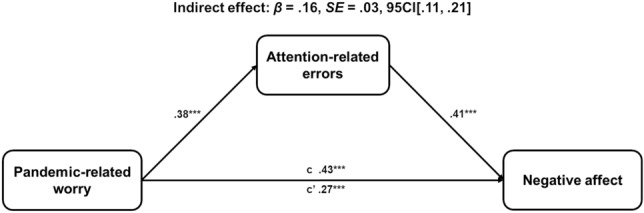
Path analysis mediation model with standardised coefficients, and indirect effect. ***p < .001

To explore whether attention-related errors mediated the relationship between PRW and negative affect, we conducted a mediation analysis using the SPSS PROCESS macro model 4 (Hayes, 2017). We entered attention-related errors (ARCES scores) as a mediator variable in the relationship between the total PRWQ score and overall negative affect (total DASS score). The rationale being that the attention-related errors would be indicative of reduced attentional resources required to regulate emotion. In order to preserve statistical power we collapsed the subscales of the DASS and the PRWQ to their total scores, however, conducting the analysis with any subscales from these measures produced the same pattern of results (Fig. 1).

We found a significant overall regression model, *R*^*2*^ = .33, *F*(1,380) = 93.07, *p* < .001, where the PRWQ score positively correlated with attention-related errors, *β* = .38, *SE* = .05, *p* < .001, 95CI[.28, .47], and attention-related errors correlated with total negative affect, *β* = .41, *SE* = .05, *p* < .001, 95CI[.32, .50]. The standardised indirect path from PRW to negative affect through attention-related errors was also significant, *β* = .16, *SE* = .03, 95CI[.11, .21]. The model showed evidence of partial mediation, as the direct relationship between PRW and negative affect remained significant even with the inclusion of the mediator, *β* = .27, *SE* = .05, *p* < .001, 95CI[.18, .36]. The total relationship between PRW and negative affect without ARCES score in the model was significant, *β* = .43, *SE* = .05, *p* < .001, 95CI[.34, .52].

In line with cognitive models of worry and anxiety (Eysenck et al., 2007; Hirsch & Mathews, 2012), the current mediation relationship could be interpreted as PRW occupying attentional capacity required to disengage from negative thoughts. Without an effective mechanism to disengage from specific PRWs, these negative thoughts would have a persistent effect on mood, elevating levels of depression, anxiety, and stress.

An alternative interpretation could be that the higher attention-related errors may have resulted in the disruption of everyday functioning leading to more stressful outcomes (e.g. missing bill payments, poorer exam grades), and that this indirectly increased negative affect. Both interpretations of the mediation relationship are not mutually exclusive and would likely interact (Moran, 2016).

As with the relationship between PRW and attention and memory errors, all paths in the mediation model remained significant when controlling for recalled trait worry and anxiety prior to the pandemic, *R*^*2*^ = .48, *F*(4, 377) = 85.69, *p* < .001. PRW still significantly correlated with attention-related errors, *β* = .24, *SE* = .05, *p* < .001, 95CI[.14, .33], and attention-related errors correlated with total negative affect, *β* = .25, *SE* = .04, *p* < .001, 95CI[.17, .34]. The standardised indirect path from PRW to negative affect through attention-related errors also remained significant when controlling for trait worry and anxiety, *β* = .06, *SE* = .02, 95CI[.03, .10], as was the direct path (c′) between PRWQ score and negative affect, *β* = .15, *SE* = .04, *p* < .001, 95CI[.07, .24].

The reason that this independent relationship is so important is because it could be indicative of an elevated risk of developing more prolonged anxious symptoms (and the development of anxiety disorders), even in individuals who previously did not experience high levels of anxiety. One of the key moderating factors which determines whether an individual goes on to develop anxious symptoms is the ability to effectively disengage from negative thoughts, which prevents the persistence of anxious states (Fox et al., 2021). For instance, in children, poorer performance on executive function tasks predicts anxiety at later timepoints, beyond their initial levels of anxiety (White et al., 2011; Zainal & Newman, 2018). Without this ability to disengage from negative automatic thoughts, these thoughts can become more habitual and more easily activated resulting in chronic anxiety and mood disorders (Watkins & Nolen-Hoeksema, 2014; Wells, 1995). Further, the general negative mood induced by these habitual thought patterns can increase negative interpretation of other ongoing problems, allowing worry to persist for even longer (Davey & Meeten, 2016; Startup & Davey, 2001). Therefore, rather than reflecting a transient increase in worry and cognitive interference, which will be alleviated once the threat of the pandemic dissipates, initially flexible concerns about the pandemic may develop into habitual worries and more entrenched anxiety disorders if not identified and addressed.

Overall, the current results are consistent with the predictions of cognitive models of anxiety/worry, whereby the attention required for the control of emotion and behaviour is disrupted by the pre occupation with task-irrelevant negative thoughts, and the impaired ability to control attention is a risk factor for the development of anxiety (Derakshan, 2020; Eysenck et al., 2007; Hirsch & Mathews, 2012; Wells, 1995). Existing cognitive models focus mainly on individuals with dispositional anxiety or worry, with limited focus on how similar mechanisms can explain the transition to more severe levels of negative affectivity when exposed to stressful situations external to the individual, independent of predetermined traits. The current investigation therefore extends these models by highlighting how the same attentional processes could plausibly also underpin the general transition to higher levels of negative affect when exposed to increased levels of worry about external situations, in this case the COVID-19 pandemic (see Songco et al., 2020 for further discussion of cognitive-emotional theories and the development of anxiety).

#### Limitations

One limitation of the current investigation was that trait anxiety and worry measures were based on recalled baseline scores. Previous evidence suggests that anxious individuals often over-estimate their previous negative emotions, consistent with a mood-congruent recall bias (Cutler et al., 1996; Safer & Keuler, 2002). The current measure of trait anxiety could therefore reflect, or be contaminated by, current levels of anxiety. If true, however, we would have expected that controlling for recalled trait anxiety would have resulted in the relationship between PRW and negative affect becoming non-significant due to shared variance, which was not the case. Additionally, when pre-pandemic baseline data was controlled for in a previous study, it was found that the relationship between poorer cognitive functioning and a single item measure of COVID-19-related anxiety remained significant, replicating our pattern of results with related non-recalled measures (Fellman et al., 2020).

To further explore whether the recalled trait anxiety and worry levels were likely to be accurate, we searched for existing published and unpublished data which contained measures of trait anxiety and worry from shortly before the pandemic, and were from similar samples to Study 2: Students from the University of Roehampton, Sussex, or KCL, recruited through random opportunity sampling. Four samples were found which matched this criteria, two were from KCL students who completed the GAD-7 and PSWQ as part of a screening process and were collected in 2017 (N = 165; Feng et al., 2019) and 2019 (N = 274; Feng et al., unpublished data). The mean levels of trait anxiety and worry from these samples were comparable with our recalled levels from this period on the same measures: The GAD-7 scores from 2017 (M = 8.07, SD = 5.01) and 2019 (M = 7.59, SD = 4.99), as well as the PSWQ scores from 2017 (M = 55.87, SD = 12.84) and 2019 (M = 56.56, SD = 14.21), were nearly identical to our mean scores on our recalled measures (i.e. recalled mean GAD-7 = 8.06; recalled mean PSWQ = 56.31).

In other published data, a sample of University of Sussex students (N = 216) recruited by Davey et al. (2022) in 2019 also reported a similar level of trait worry to our sample (M = 57.39, SD = 9.87). Further, a sample of University of Roehampton students (N = 546) recruited between 2017 and 2018 by Norbury and Evans (2019) reported levels of anxiety on the Trait Anxiety Inventory (Speilberger et al., 1983) in the upper levels of the mild to moderate range, i.e. 40–50 (47.37; SD = 10.9; Van Dam et al., 2013). Consistent with our measure of trait anxiety reflecting accurate recall of pre-pandemic anxiety, our sample also reported anxiety in the higher end of the mild to moderate range on the GAD-7 (moderate anxiety score criteria = 5–9; Spitzer et al., 2006). Thus, from across four samples drawn from the identical population we recruited from, our measures of recalled trait anxiety and worry were equivalent to actual scores from the pre-pandemic period. Our measures of pre-pandemic trait anxiety and worry are therefore likely valid, despite potential recall bias.

When interpreting the current findings, we must account for the cross-sectional design, which limits the ability to draw strong conclusions about causal relationships between factors. Indeed, it is possible that negative affect results in an increase PRW, rather than the opposite relationship in the current model. Though difficult to reliably separate the true direction of effects out statistically, we conducted a reverse mediation analysis, whereby the variables were reversed in the mediation model (PRW → Negative affect → attention-related errors). This revealed that the opposite pattern of results was also found, as the indirect effect of PRW on attention-related errors mediated through negative affect was significant (whilst controlling for recalled trait anxiety and worry), *β* = .08, *SE* = .02, 95CI[.05, .12]. The results therefore suggest that the opposite interpretation remains plausible. Indeed, the data could reflect a bidirectional relationship, where worry’s impact on negative affect could reduce attentional control, concurrent with worry’s deleterious effect on attentional control increasing negative affect through the persistence of worry episodes – consistent with a self-perpetuating relationship (Eysenck et al., 2007; Hotton et al., 2018; Moran, 2016). We note that the reverse mediation method does not always reliably detect the ‘true’ direction of causality (Lemmer & Gollwitzer, 2017; Thoemmes, 2015). Future investigations exploring the lasting effects of PRW on cognition and mental health should therefore utilise a longitudinal design, as this would allow the inference about directional relationships. Here, however, we present a plausible theoretically grounded model of how PRW may disrupt cognitive function, especially attentional capacity, and how this may result in elevated negative affect.

Additionally, when investigating the enduring effect of PRW on cognitive function, more objective task-based behavioural measures could be used. These could identify specific executive functions which are most susceptible to interference from PRW, such as the impact on inhibition or shifting (Mennies et al., 2021). However, using more sophisticated task-based measures would require larger sample sizes to account for potentially lower statistical power in such studies (Hedge et al., 2018). Specific moderating risk factors not explored in the current investigation could also be explored in relation to persistent PRW in later stages of the pandemic, for instance, the death of a loved one due to COVID-19 or redundancy due to the pandemic (Blustein & Guarino, 2020; Torrens-Burton et al., 2022).

#### Implications

The current investigation provides key findings to show that, firstly, the PRWQ can act as a suitable validated measure to explore enduring levels of PRW, and could be used as a measure to assess risk of developing lasting anxious symptoms in response to the pandemic. Indeed, whilst not every individual may develop lasting anxiety, a substantial percentage may experience prolonged anxious symptoms (Shevlin et al, 2021). Importantly, our preliminary study has shown that the key factor that most strongly correlated with cognitive functioning is decline in QoL during the pandemic—which is likely to last beyond the peak of the pandemic, due to economic impacts. Interventions aiming to improve mental health both during and after the pandemic should therefore target this PRW specifically, especially in young adults. Evidence has suggested that CBT targeting fears about infection from COVID-19 were successful in reducing anxiety in the initial stages of the pandemic (Wahlund et al., 2021), meaning that these novel worries may be sensitive to similar treatments.

## Conclusions

The current results suggest that PRW may not just reflect the preoccupation with direct threat from COVID-19, but is a multifaceted construct encompassing broader concerns about the indirect impact of the pandemic to physical and mental health, social life, academic achievement, and financial concerns. Further, these PRWs appear to correlate with poorer cognitive functioning, especially attention-related errors. Even when controlling for recalled trait anxiety and worry we found a significant relationship, indicative of an independent effect across the sample even in those without pre-existing severe anxiety. Further, the relationship between PRW and negative affect was partially mediated through attention-related errors, consistent with the PRW occupying the attentional-capacity required to regulate negative emotions in the face of an array of novel pandemic-related stressors. Based on the current evidence, worries about pandemic-specific concerns may be a key factor in the decline in mental health, which need to be addressed in order to support psychological recovery and resilience in the wake of the pandemic.

### Electronic supplementary material

Below is the link to the electronic supplementary material.

Supplementary file1 (DOCX 49 kb)

Supplementary file2 (DOCX 20 kb)

Supplementary file3 (DOCX 34 kb)

## Data Availability

OSF open data link: https://osf.io/k8qna/?view_only=6a7dc519471349afb2eb60df5a7b9bcc.

## Appendix

### Pandemic-Related Worry Questionnaire—20 item Version

Please answer the questions below on a scale of 1–5 ranging from ‘Strongly disagree’ to ‘Strongly agree’. Please answer each statement for how you have felt OVER THE PAST MONTH.

In this case we refer to worry as an automatic negative thought which persistently occupies your attention. Don’t take too much time to answer the questions, and respond based on how you actually feel rather than any other reference (e.g. how you think you should feel).

Please answer along the five-point scale: 1 = Strongly disagree 2 = Disagree 3 = Neither agree nor disagree 4 = Agree 5 = Strongly agree

**Table:**

Previous Item number	New item number	Subscale	Item
1	1	Infection probability	I often worry about the possibility that I have COVID-19
2	2	Infection probability	I get concerned when I experience symptoms in case I have COVID-19
3	3	Infection probability	When I learn or read about COVID-19 I become worried that I may have it
4	4	Infection probability	If COVID-19 is brought to my attention (through the radio, television, newspapers, or someone I know), I worry about getting it myself
5	5	Risk to loved ones	I often worry about my family members getting COVID-191
7	6	Financial concerns	Hearing about job losses in the media or through people I know makes me worry about my job security
8	7	Financial concerns	I often worry about the impact of COVID-19 on my financial situation
9	8	Decline in QoL	I often think about the impact of social distancing and how this will affect me
11	9	Risk to loved ones	I often worry that someone I love (e.g., family, close friends) will get infected by COVID-19 virus
12	10	Financial concerns	I often worry about the impact of COVID-19 on my family’s financial situation
18	11	Decline in QoL	I often worry that my work/academic performance will be impacted due to COVID -19 pandemic
19	12	Decline in QoL	I often worry that my relationships will be impacted due to COVID -19 pandemic
20	13	Infection severity	I often worry that if I get COVID -19 I will not recover from it
21	14	Risk to loved ones	I often worry that if my close friend(s) or family member(s) contracted COVID -19, they will not recover from it
22	15	Infection severity	I often worry that I will get hospitalised or will die due to COVID -19
23	16	Risk to loved ones	I often worry about whether my close friend(s) or family member(s) will be hospitalised or die due to COVID -19
24	17	Decline in QoL	I often worry that the government Covid-19 rules (e.g. limited socialising) will influence my own physical and/or mental health
26	18	Decline in QoL	I often worry that things will not get back to normal
28	19	Decline in QoL	I often worry that I am not performing well in work/study during the government Covid-19 rules (e.g. limited socialising)
30	20	Decline in QoL	I often worry that the isolation situation will influence my relationship with people I live with in a bad way

## Footnotes

1 Despite the wide age range, the sample was composed of mainly young adults, with 91.88% of participants being 25 or lower (N = 351). Exclusion of those over 25 did not alter the significance or pattern of results. All participants were therefore retained due to no a priori exclusion criteria for age; however, caution should be taken when generalising the current findings to older participants.

2 The inclusion of COVID-19 infection as a binary covariate did not alter the pattern or significance of any of the results, thus excluding the possibility that relationships found reflected the direct impact of COVID-19 on cognitive function, rather than PRW (e.g. Hampshire et al., 2021).
