# Supplementary material for: Specific Pandemic-Related Worries Predict Higher Attention-Related Errors and Negative Affect Independent of Trait Anxiety in UK-Based Students
Source: Cognit Ther Res. 2022 Oct 20;47(1):1–19. doi: 10.1007/s10608-022-10336-7 (PMC9584227; doi:10.1007/s10608-022-10336-7)
Supplement: Supplementary file 1 — Supplementary file1 (DOCX 49 kb) [file 10608_2022_10336_MOESM1_ESM.docx]

**Supplementary Materials 1**


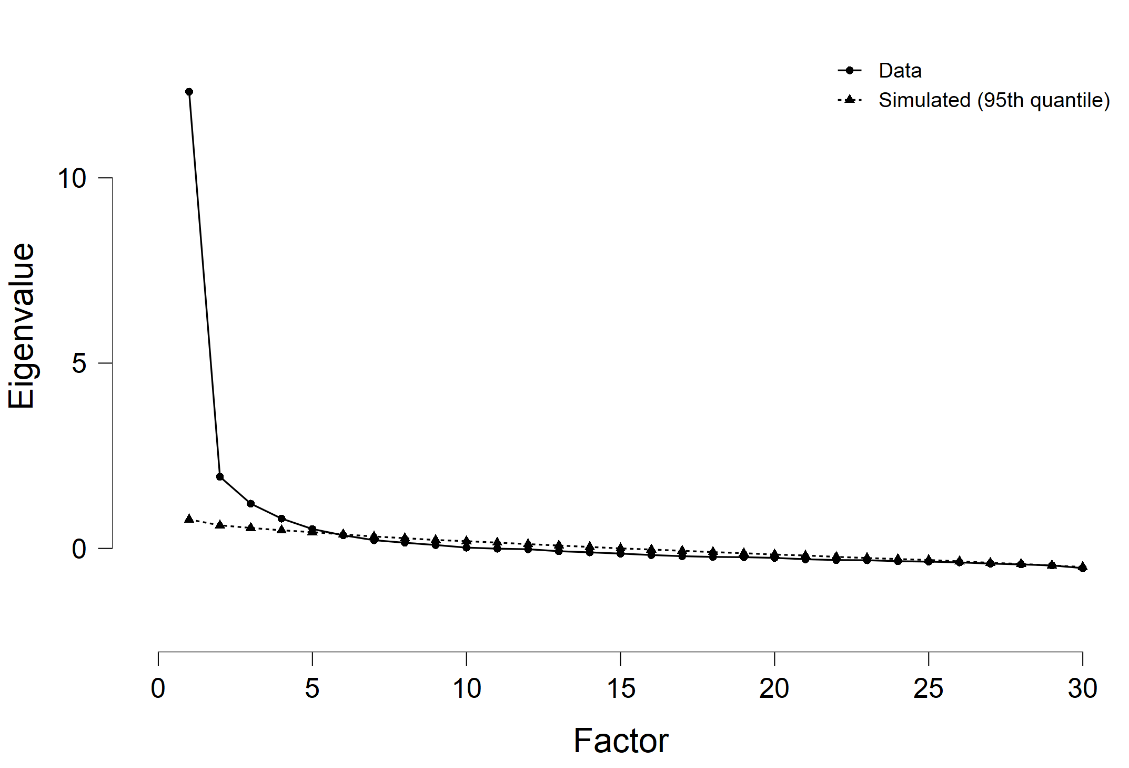


Figure S1. Scree plot presenting eigenvalues with simulated 95^th^ quantile eigenvalues overlaid.
