# Supplementary material for: Specific Pandemic-Related Worries Predict Higher Attention-Related Errors and Negative Affect Independent of Trait Anxiety in UK-Based Students
Source: Cognit Ther Res. 2022 Oct 20;47(1):1–19. doi: 10.1007/s10608-022-10336-7 (PMC9584227; doi:10.1007/s10608-022-10336-7)
Supplement: Supplementary file 2 — Supplementary file2 (DOCX 20 kb) [file 10608_2022_10336_MOESM2_ESM.docx]

| Item number | Item | Personal COVID-19 risk | Decline in QoL | Family COVID-19 risk | Financial concerns |
| --- | --- | --- | --- | --- | --- |
| **20** | **I often worry that if I get COVID -19 I will not recover from it** | **0.77** |  |  |  |
| **1** | **I often worry about the possibility that I have COVID-19** | **0.766** |  |  |  |
| **22** | **often worry that I will get hospitalised or will die due to COVID -19** | **0.755** |  |  |  |
| **3** | **When I learn or read about COVID-19 I become worried that I may have it** | **0.729** |  |  |  |
| **10** | **I often worry that I will get infected by COVID-19 virus** | **0.728** |  |  |  |
| **4** | **If COVID-19 is brought to my attention (through the radio, television, newspapers, or someone I know), I worry about getting it myself** | **0.644** |  |  |  |
| **2** | **I get concerned when I experience symptoms in case I have COVID-19** | **0.574** |  | 0.221 |  |
| 17 | I often worry that I will not get everyday necessities like food and other grocery items due to COVID-19 pandemic | 0.451 | 0.248 |  |  |
| 13 | I often worry about the consequence of COVID-19 for myself | 0.432 | 0.269 |  | 0.264 |
| 27 | I often worry that I am not taking the right actions to prevent the spread of COVID -19 | 0.2 | 0.401 |  |  |
| **24** | **I often worry that the lock down will influence my own physical and/or mental health** |  | **0.776** |  |  |
| **30** | **I often worry that the isolation situation will influence my relationship with people I live with in a bad way** |  | **0.691** |  |  |
| **26** | **I often worry that things will not get back to normal** |  | **0.635** |  |  |
| **9** | **I often think about the impact of social distancing and how this will affect me** |  | **0.598** | 0.255 |  |
| **19** | **I often worry that my relationships will be impacted due to COVID -19 pandemic** |  | **0.577** |  |  |
| 25 | I often worry that the lock down will influence the physical and/or mental health of someone I am close to (e.g., family, close friends) |  | 0.554 | 0.28 |  |
| **18** | **I often worry that my work/academic performance will be impacted due to COVID -19 pandemic** |  | **0.53** |  |  |
| **28** | **I often worry that I am not performing well in work/study during the lock down** |  | **0.525** |  |  |
| 16 | I often worry that I will not achieve something important to me due to COVID-19 pandemic |  | 0.37 |  | 0.341 |
| 15 | I often worry about the consequence of COVID-19 for my close friend |  | 0.353 | 0.26 |  |
| 6 | The COVID-19 pandemic makes me worry about the future. |  | 0.32 | 0.28 | 0.325 |
| 29 | I often worry that my future job/study prospects will be influenced by COVID - 19 pandemic |  | 0.32 |  | 0.482 |
| 14 | I often worry about the consequence of COVID-19 for my family |  | 0.258 | 0.54 |  |
| **5** | **I often worry about my family members getting COVID-19** |  |  | **0.859** |  |
| **21** | **I often worry that if my close friend(s) or family member(s) contracted COVID -19, they will not recover from it** |  |  | **0.836** |  |
| **11** | **I often worry that someone I love (e.g., family, close friends) will get infected by COVID-19 virus** |  |  | **0.812** |  |
| **23** | **I often worry about whether my close friend(s) or family member(s) will be hospitalised or die due to COVID -19** |  |  | **0.748** |  |
| **7** | **Hearing about job losses in the media or through people I know makes me worry about my job security.** |  |  |  | **0.821** |
| **8** | **I often worry about the impact of COVID-19 on my financial situation** |  |  |  | **0.775** |
| **12** | **I often worry about the impact of COVID-19 on my family’s financial situation** |  |  |  | **0.489** |
|  | Eigenvalues | 12.88 | 2.55 | 1.76 | 1.45 |
|  | Percentage variance | 42.95 | 8.50 | 5.98 | 4.82 |
|  | Cronbach’s α | .913 | .853 | .923 | .834 |
|  | Selected Items | 7 | 7 | 4 | 3 |
|  | Selected item mean response (SD) | 2.63 (1.19) | 3.50 (1.21) | 3.78 (1.13) | 3.34 (1.31) |

**Supplementary materials 2**

**Four factor model**

Table S1. List of all items loading on four factors derived from the Exploratory Factor Analysis (*N* = 255). Item factor loadings are highlighted in grey and bold. Only loadings with an absolute value greater than .2 are displayed for visibility.
