# Supplementary material for: Specific Pandemic-Related Worries Predict Higher Attention-Related Errors and Negative Affect Independent of Trait Anxiety in UK-Based Students
Source: Cognit Ther Res. 2022 Oct 20;47(1):1–19. doi: 10.1007/s10608-022-10336-7 (PMC9584227; doi:10.1007/s10608-022-10336-7)
Supplement: Supplementary file 3 — Supplementary file3 (DOCX 34 kb) [file 10608_2022_10336_MOESM3_ESM.docx]

**Supplementary materials 3**

**Bootstrapped dominance analysis**

To explore the relative importance of each Pandemic Related Worry Questionnaire (PRWQ) subscale in predicting attention and memory-related errors, we ran a dominance analysis using the *dominanceanalysis* R package (Navarrete & Soares, 2020). Each PRWQ subscale was entered as a predictor in the model, with attention-related errors entered as the outcome variable in one analysis, and memory-related errors as the outcome in another analysis. To assess the reproducibility of the dominance structure, we ran 5000 bootstrapped samples for these two analyses. The results are listed in the two tables below.

**Bootstrapped Dominance Analysis: Table interpretation**

In the table ‘Dominance’ refers to the form of dominance computed, depending on the relative inclusion of other predictors within the model. Complete dominance reflects the dominance of one variable over another with all other predictors included in the model. In the current investigation, interpretations are based on complete dominance (see Budescu, 1993; Azen & Budescu, 2003; Navarrete & Soares, 2020, for further guidance on conducting and interpreting dominance analysis).

The columns i and j report the contrasted pairs of predictors, the header Dij reports the qualitative level of dominance achieved overall, with the value of 1 reflecting i dominating j, and 0 reflects j dominating i, whilst a value of .5 reflects no clear pattern of dominance. mDij reflects the mean level of dominance i achieved over j across bootstrapped samples, with SE.Dij reflecting the standard error of this dominance. Pij reflects the proportion of samples that i dominated j, whilst Pji reflects the opposite pattern of dominance, when j dominates i. The Pnoij column reports the proportion of bootstrapped samples where no clear pattern of dominance was reached between i and j. The reproducibility column reflects the proportion of bootstrapped samples that the pattern of dominance between i-j is found, regardless of the outcome (i.e. i dominating j, j dominating i, or no dominance being found).

**Bootstrapped dominance analysis: Attention-related errors**

Table S1. Results from the bootstrapped dominance analysis across 5000 samples with all Pandemic-Related Worry Questionnaire subscales as predictors, and Attention Related Cognitive Errors Scale score as outcome variable.

| Dominance | i | j | Dij | mDij | SE.Dij | Pij | Pji | Pnoij | Reproducibility |
| --- | --- | --- | --- | --- | --- | --- | --- | --- | --- |
| complete | DeclineQoL | COVIDprobability | 1 | .886 | .224 | .784 | .012 | .203 | .784 |
| complete | DeclineQoL | COVIDseverity | 1 | .963 | .137 | .929 | .003 | .067 | .929 |
| complete | DeclineQoL | RiskToLovedOnes | 1 | .985 | .084 | .971 | 0 | .029 | .971 |
| complete | DeclineQoL | FinancialConcerns | 1 | .996 | .053 | .993 | .001 | .006 | .993 |
| complete | COVIDprobability | COVIDseverity | 1 | .735 | .384 | .639 | .17 | .192 | .639 |
| complete | COVIDprobability | RiskToLovedOnes | 1 | .817 | .281 | .675 | .042 | .283 | .675 |
| complete | COVIDprobability | FinancialConcerns | 1 | .729 | .266 | .474 | .017 | .509 | .474 |
| complete | COVIDseverity | RiskToLovedOnes | .5 | .647 | .309 | .381 | .087 | .533 | .533 |
| complete | COVIDseverity | FinancialConcerns | .5 | .552 | .188 | .128 | .024 | .848 | .848 |
| complete | RiskToLovedOnes | FinancialConcerns | .5 | .491 | .205 | .075 | .093 | .832 | .832 |
| conditional | DeclineQoL | COVIDprobability | 1 | .937 | .207 | .904 | .031 | .065 | .904 |
| conditional | DeclineQoL | COVIDseverity | 1 | .982 | .111 | .972 | .008 | .020 | .972 |
| conditional | DeclineQoL | RiskToLovedOnes | 1 | .992 | .064 | .985 | .001 | .015 | .985 |
| conditional | DeclineQoL | FinancialConcerns | 1 | .996 | .057 | .994 | .002 | .004 | .994 |
| conditional | COVIDprobability | COVIDseverity | 1 | .721 | .412 | .658 | .216 | .125 | .658 |
| conditional | COVIDprobability | RiskToLovedOnes | 1 | .84 | .299 | .75 | .070 | .18 | .75 |
| conditional | COVIDprobability | FinancialConcerns | 1 | .813 | .299 | .687 | .061 | .252 | .687 |
| conditional | COVIDseverity | RiskToLovedOnes | 1 | .66 | .351 | .458 | .137 | .405 | .458 |
| conditional | COVIDseverity | FinancialConcerns | .5 | .629 | .3675 | .433 | .174 | .393 | .393 |
| conditional | RiskToLovedOnes | FinancialConcerns | .5 | .455 | .31 | .15 | .241 | .608 | .608 |
| general | DeclineQoL | COVIDprobability | 1 | .945 | .228 | .945 | .055 | 0 | .945 |
| general | DeclineQoL | COVIDseverity | 1 | .985 | .121 | .985 | .015 | 0 | .985 |
| general | DeclineQoL | RiskToLovedOnes | 1 | .996 | .06 | .996 | .004 | 0 | .996 |
| general | DeclineQoL | FinancialConcerns | 1 | .997 | .058 | .997 | .003 | 0 | .997 |
| general | COVIDprobability | COVIDseverity | 1 | .738 | .44 | .738 | .262 | 0 | .738 |
| general | COVIDprobability | RiskToLovedOnes | 1 | .852 | .355 | .852 | .148 | 0 | .852 |
| general | COVIDprobability | FinancialConcerns | 1 | .78 | .414 | .78 | .22 | 0 | .78 |
| general | COVIDseverity | RiskToLovedOnes | 1 | .644 | .479 | .644 | .356 | 0 | .644 |
| general | COVIDseverity | FinancialConcerns | 1 | .553 | .497 | .553 | .447 | 0 | .553 |
| general | RiskToLovedOnes | FinancialConcerns | 0 | .364 | .481 | .364 | .636 | 0 | .636 |

**Bootstrapped dominance analysis: Memory related errors**

Table S2. Results from the bootstrapped dominance analysis across 5000 samples with all Pandemic-Related Worry Questionnaire subscales as predictors, and Memory Failures Scale as outcome variable.

| Dominance | i | j | Dij | mDij | SE.Dij | Pij | Pji | Pnoij | Reproducibility |
| --- | --- | --- | --- | --- | --- | --- | --- | --- | --- |
| complete | DeclineQoL | COVIDprobability | 0 | .293 | .345 | .117 | .531 | .351 | .531 |
| complete | DeclineQoL | COVIDseverity | 1 | .721 | .308 | .509 | .067 | .424 | .509 |
| complete | DeclineQoL | RiskToLovedOnes | 1 | .83 | .265 | .687 | .028 | .286 | .687 |
| complete | DeclineQoL | FinancialConcerns | 1 | .929 | .213 | .887 | .03 | .083 | .887 |
| complete | COVIDprobability | COVIDseverity | 1 | .931 | .224 | .902 | .041 | .057 | .902 |
| complete | COVIDprobability | RiskToLovedOnes | 1 | .968 | .14 | .946 | .009 | .045 | .946 |
| complete | COVIDprobability | FinancialConcerns | 1 | .95 | .154 | .901 | .002 | .097 | .901 |
| complete | COVIDseverity | RiskToLovedOnes | 1 | .644 | .323 | .394 | .106 | .501 | .394 |
| complete | COVIDseverity | FinancialConcerns | .5 | .61 | .226 | .236 | .016 | .748 | .748 |
| complete | RiskToLovedOnes | FinancialConcerns | .5 | .515 | .221 | .113 | .082 | .804 | .804 |
| conditional | DeclineQoL | COVIDprobability | 0 | .275 | .4 | .196 | .646 | .158 | .646 |
| conditional | DeclineQoL | COVIDseverity | 1 | .761 | .342 | .63 | .109 | .261 | .63 |
| conditional | DeclineQoL | RiskToLovedOnes | 1 | .853 | .267 | .745 | .039 | .216 | .745 |
| conditional | DeclineQoL | FinancialConcerns | 1 | .926 | .23 | .895 | .042 | .063 | .895 |
| conditional | COVIDprobability | COVIDseverity | 1 | .932 | .234 | .914 | .050 | .035 | .914 |
| conditional | COVIDprobability | RiskToLovedOnes | 1 | .973 | .142 | .959 | .014 | .027 | .959 |
| conditional | COVIDprobability | FinancialConcerns | 1 | .975 | .13 | .959 | .01 | .031 | .959 |
| conditional | COVIDseverity | RiskToLovedOnes | 1 | .662 | .351 | .461 | .137 | .402 | .461 |
| conditional | COVIDseverity | FinancialConcerns | 1 | .706 | .366 | .558 | .147 | .295 | .558 |
| conditional | RiskToLovedOnes | FinancialConcerns | .5 | .522 | .301 | .204 | .16 | .636 | .636 |
| general | DeclineQoL | COVIDprobability | 0 | .259 | .438 | .259 | .741 | 0 | .741 |
| general | DeclineQoL | COVIDseverity | 1 | .738 | .44 | .738 | .262 | 0 | .738 |
| general | DeclineQoL | RiskToLovedOnes | 1 | .869 | .337 | .869 | .131 | 0 | .869 |
| general | DeclineQoL | FinancialConcerns | 1 | .939 | .24 | .939 | .061 | 0 | .939 |
| general | COVIDprobability | COVIDseverity | 1 | .938 | .242 | .938 | .062 | 0 | .938 |
| general | COVIDprobability | RiskToLovedOnes | 1 | .977 | .149 | .977 | .023 | 0 | .977 |
| general | COVIDprobability | FinancialConcerns | 1 | .975 | .156 | .975 | .025 | 0 | .975 |
| general | COVIDseverity | RiskToLovedOnes | 1 | .709 | .454 | .709 | .291 | 0 | .709 |
| general | COVIDseverity | FinancialConcerns | 1 | .749 | .434 | .749 | .251 | 0 | .749 |
| general | RiskToLovedOnes | FinancialConcerns | 1 | .56 | .496 | .56 | .440 | 0 | .56 |

**References**

Azen, R., & Budescu, D. V. (2003). The dominance analysis approach for comparing predictors in multiple regression. Psychological Methods, 8(2), 129–148.

Budescu, D. V. (1993). Dominance analysis: A new approach to the problem of relative importance of predictors in multiple regression. Psychological Bulletin, 114(3), 542–551.

Navarrete, C. B., & Soares, F. C. (2020). dominanceanalysis: Dominance analysis (2.0.0) [Computer software]. <https://CRAN.R-project.org/package=dominanceanalysis>
